# Different effects of femoral and tibial rotation on the different measurements of patella tilting: An axial computed tomography study

**DOI:** 10.1186/1749-799X-3-5

**Published:** 2008-02-12

**Authors:** Yeong-Fwu Lin, Mei-Hwa Jan, Da-Hon Lin, Cheng-Kung Cheng

**Affiliations:** 1 Institute of Biomedical Engineering, National Yang Ming University. No. 155, Sec 2, Li-Nung Street, Taipei 112, Taiwan; 2 Department of Orthopaedics. West Garden Hospital. No. 270, Sec 2, Siyuan Road, Taipei 108, Taiwan; 3 School and Graduate Institute of Physical Therapy, College of Medicine, National Taiwan University. No. 17, XuZhou Road, Taipei 100, Taiwan; 4 Department of Orthopaedics, En Chu Kong Hospital. No. 399, Fu-Hsin Road, Sang Shia, Taipei County 237, Taiwan

## Abstract

**Background:**

The various measurements of patellar tilting failed to isolate patellar tilting from the confounding effect of its neighboring bone rotation (femoral and tibial rotation) in people sustaining patellofemoral pain (PFPS). Abnormal motions of the tibia and the femur are believed to have an effect on patellofemoral mechanics and therefore PFPS. The current work is to explore the various effects of neighboring bone rotation on the various measurements of patellar tilting, through an axial computed tomography study, to help selecting a better parameter for patella tilting and implement a rationale for the necessary intervention at controlling the limb alignment in the therapeutic regime of PFPS.

**Methods:**

Forty seven patients (90 knees), comprising of 34 females and 11 males, participated in this study. Forty five knees, from randomly selected sides of bilaterally painful knees and the painful knees of unilaterally painful knees, were enrolled into the study. From the axial CT images in the subject knees in extension with quadriceps relaxed, the measurements of femoral rotation, tibial rotation, femoral rotation relative to tibia, and 3 parameters for patella tilting were obtained and analyzed to explore the relationship between the different measurements of patella tilt angle and the measurements of its neighboring bone rotation (femoral, tibial rotation, and femoral rotation relative to tibia).

**Results:**

The effect of femoral, tibial rotation, and femoral rotation relative to tibia on patella tilting varied with the difference in the way of measuring the patella tilt angle. Patella tilt angle of Grelsamer increased with increase in femoral rotation, and tibial rotation. Patella tilt angle of Sasaki was stationary with change in femoral rotation, tibial rotation, or femoral rotation relative to tibia. While, modified patella tilt angle of Fulkerson decreased with increase in femoral rotation, tibial rotation, or femoral rotation relative to tibia.

**Conclusion:**

The current study has demonstrated various effects of regional bony alignment on the different measurements of the patellar tilt. And the influence of bony malalignment on the patellar tilt might draw a clinical implication that patellar malalignment can not be treated, separately, independent of the related limb alignment. This clinical implication has to be verified by further works, with a comprehensive evaluation of the various treatments of patellar malalignment.

## Background

Patellofemoral pain is a common affliction, caused by a large variety of factors. Patients with patellofemoral pain syndrome (PFPS) present one of the most substantial diagnostic and therapeutic challenges to orthopedic surgeons worldwide [1]. The etiology of PFPS mainly lies in a disorder of the patella tracking. Recently reports declared that any assertion of a link existing or not between patellar malalignment and PFPS is based on assumption, not evidence.[2] There exists a large body of evidence indicating that radiological measures of patellar malalignment and symptoms of PFPS are poorly correlated. As thus contrary to popular belief, the existence of patellar malalignment in subjects with PFPS is uncertain or suggests otherwise.[2-11] However, these current evidences are based largely upon measurement techniques that demonstrate poor reliability and/or validity. The true amount of lateral patellar displacement has been verified to be overestimated.[12] In the long run the fault might be proved to be on the measure, not of the theory it self.[2,12]

Femoral internal rotation has been demonstrated to be the primary contributor to lateral patellar tilt. [13,14] Currently the various measurements of patellar tilting failed to isolate patellar tilting from the confounding effect of its neighboring bone rotation (femoral and tibial rotation) in people sustaining patellofemoral pain (PFPS). Abnormal motions of the tibia and femur are believed to have an effect on patellofemoral mechanics and therefore PFPS. [13] The current work is to explore the various effects of neighboring bone rotation on the various measurements of patellar tilting, through an axial computed tomography study, to help selecting a better measurement for patella tilting and implement a rationale for the necessary intervention at controlling the limb alignment in the therapeutic regime of PFPS. We hypothesized that the neighboring bone rotation (femoral and tibial) around the knee might exert different effects on different measurements of patellar tilting.

## Methods

### Patient selection

Patient selection was based on the following inclusion criteria: 1. Each patient's pain originated from the patellofemoral joint; 2. Patellar pain for at least 3 months; 3. Pain when performing at least three of the following knee-flexing activities: sitting, standing from a prolonged sitting, stair ascent or descent, squatting, running, kneeling, or jumping; 4. Presence of pain or crepitation during patella grinding test, or positive apprehension test. Exclusion criteria included the presence of any major medical disease, rheumatoid arthritis, gouty arthritis, image findings of osteoarthritis, patellar tendonitis, meniscal injury or other internal derangement of the knee, patellar dislocation, frank laxity or ligamentous instability of the knee, varus or valgus deformity of the knee, previous knee surgery, spinal or hip referred pain, or leg length discrepancy.

Forty seven patients (90 knees), comprising of 34 females and 11 males, participated in this study. All signed an informed consent approved by the Ethics Committee of the author's hospital. The mean patient age was 38.0 ± 9.59 years, ranging from 18 to 50 years. Twelve individuals suffered unilateral PFPS, while 33 had bilateral PFPS. Therefore there were 78 painful and 12 pain free knees investigated in this study. The randomized selected sides of bilaterally painful knees and the painful knees of unilaterally painful knees were sampled for data analysis, comprising a total of 45 subject knees.

### CT imaging

All patients were examined with axial computed tomography on both knees in extension, with the quadriceps relaxed as well as contracted according to Gigante's methods [15]. The subject was placed in the supine position and a series of axial CT images of 5 mm slice thickness were obtained with a Pace General Electric CT machine (GE Medical Systems, Milwaukee, WI). Scans were obtained with knees in extension with quadriceps relaxed. Both feet were fastened together with a Velcro strap to avoid external rotation of both legs. An axial image at the widest diameter of the patella was used for the measurement [15]. To enhance reproducibility, all measurements were made using Centricity radiology RA 600 image software (version 6.1, GE Medical Systems, Milwaukee, WI). The inter-reliability of measurement for various parameters between two observers ranged from 0.80 to 0.91.

### CT measurements of patellar alignments

**Figure 1:**
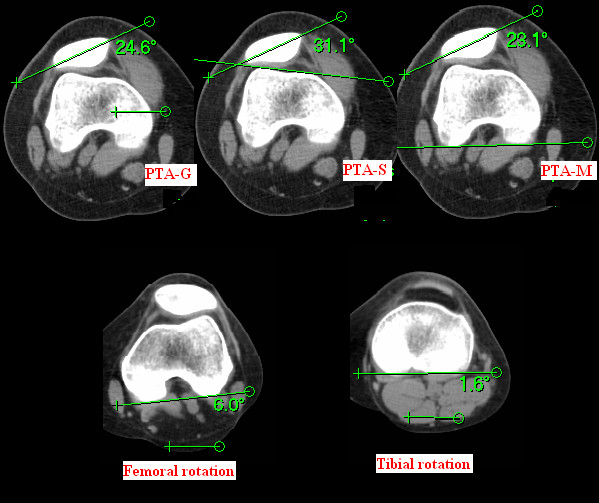
Measurements of PTAs and the neighboring bone rotation of the knee. PTA-G: patella tilt angle of Grelsamer; PTA-S: patella tilt angle of Sasaki; PTA-M: modified patella tilt angle of Fulkerson; FR: femoral rotation; and TR: tibial rotation.

The following measurements were obtained: 1) patella tilt angle of Grelsamer (PTA-G, the angle subtended by a line joining the medial and lateral edges of the patella and the horizontal) [16], 2) patella tilt angle of Sasaki (PTA-S, the angle sustended by a line through the medial and lateral edge of the patella and another line through the anterior border of both femoral condyles) [17], 3) patella tilt angle of Fulkerson (PTA-M, The angle subtended by a line joining the medial and lateral edges of the patella and a line drawn along the posterior femoral condyles)[18], 4) femoral rotation (FR, the angle sustended by a line drawn through the two most posterior points of the posterior femoral condyles and the horizontal, with plus as external rotation, and minus as internal rotation), and 5) tibial rotation (TR, the angle subtended by a line drawn through the two most posterior points along the posterior border of the proximal tibia and the horizontal, with plus as external rotation, and minus as internal rotation).(Figure 1) 6) femoral rotation relative to tibia (FRRT, the angle computed from "FR-TR", with plus as external rotation, and minus as internal rotation). As thus axial computed tomography images of patients with PFPS were analyzed to explore the relationship between the different measurements of patella tilt angle and the measurements of its neighboring bone rotation (FR, TR, and FRRT).

### Statistical analysis

A Kolmogorov-Smirnoy normality test (SPSS version 11, SPSS Inc, Chicago, IL) confirmed that all variables were normally distributed. Pearson correlation and regression analysis by curve estimation was preformed to demonstrate the association between the measurements of patella tilting and the measurements of femoral, tibial rotation, or femoral rotation relative to tibia, and to trace whether the 3 different patella tilt angle measurements were affected by femoral or tibial rotation. Differences were considered to be significant when *p *< 0.05.

#### Ethical Board Review statement

Each author certifies that his or her institution has approval the human protocol for this investigation and that all investigations were conducted in conformity with ethical principles of research, and the informed consent was obtained.

## Results

**Figure 2:**
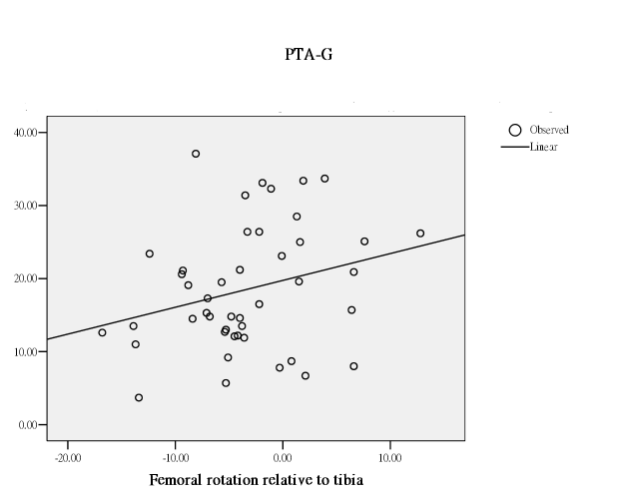
Through regression analysis, curve estimation of the predictability of femoral rotation relative to tibia, as an independent variable, in serving as an explanatory predictor of PTA-G, as dependent variables. PTA-G increased with increase in femoral rotation relative to tibia.

**Figure 3:**
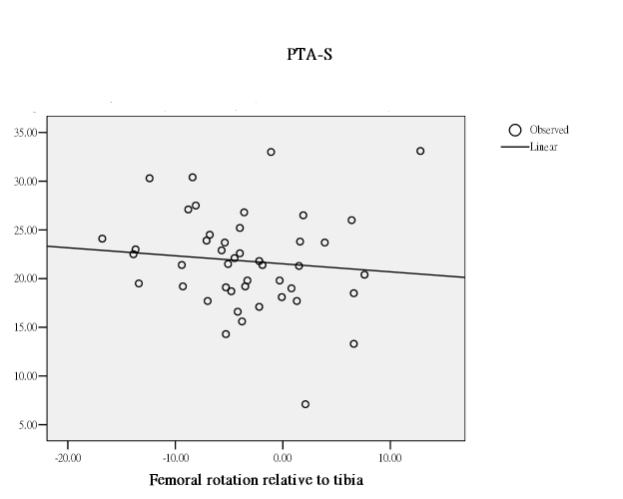
Through regression analysis, curve estimation of the predictability of femoral rotation relative to tibia, as an independent variable, in serving as an explanatory predictor of PTA-S, as dependent variables. PTA-S was stationary with increase in femoral rotation relative to tibia.

**Figure 4:**
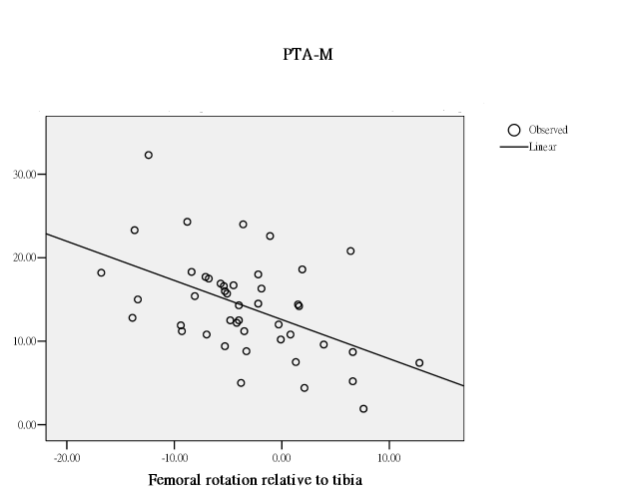
Through regression analysis, curve estimation of the predictability of femoral rotation relative to tibia, as an independent variable, in serving as an explanatory predictor of PTA-M, as dependent variables. PTA-M decreased with increase in femoral rotation relative to tibia.

The effect of femoral rotation, tibial rotation or femoral rotation relative to tibia on patella tilting varied with the difference in the way of measuring the patella tilt angle. (Table 1) All rotation related measurements rendered a different effect on the 3 different measurements of patella tilt angle. (Table 1) PTA-G increased with increase in external femoral rotation, increase in external tibial rotation, and increase in femoral rotation relative to tibia. PTA-S was stationary with increase in external femoral rotation, increase in external tibial rotation, and increase in femoral rotation relative to tibia. In contrast, PTA-M decreased with increase in external femoral rotation, increase in external tibial rotation, and increase in femoral rotation relative to tibia. (Figure 2, 3, 4)

The measurements of femoral rotation, tibial rotation, and femoral rotation relative to tibia, and patella tilt angle, PTA-G, PTA-S, and PTA-M were presented in Table 1. Deserving special mention was that the 95% confidence interval of PTA-S was more focused on its mean. We are not trying to overstate the probable implication, but it might address some concern about PTA-S in better serving as a parameter of rotational patellar alignment.

**Table 1:** Measurements of patella tilt angles and its neighboring bone rotation

	Mean ± SD (N = 45)	95% CI for Mean	Minimum	Maximum
Bone rotation				
FR	5.10 ± 10.52	1.94~8.26	-12.20	24.80
TR	8.48 ± 8.27	6.00~10.97	-8.40	29.90
FRRT	-3.38 ± 6.21	-3.17~3.85	-16.80	12.80
Patellar alignment				
PTA-G	18.51 ± 8.46	15.97~21.05	3.70	37.10
PTA-S	21.80 ± 5.03	20.28~23.31	7.10	33.10
PTA-M	14.17 ± 5.90	12.40~15.94	1.90	32.30

CI: Confidence Interval for Mean; FR: Femoral rotation; TR: Tibial rotation; FRRT: Femoral rotation relative to tibia; PTA-G: Patella tilt angle of Grelsamer; PTA-S: Patella tilt angle of Sasaki; PTA-M: modified patella tilt angle of Fukerson.

PTA-G was highly correlated with femoral rotation, and tibial rotation. (*p *< 0.01). PTA-M was highly correlated with femoral rotation, and femoral rotation relative to tibia (*p *< 0.01), and moderately correlated tibial rotation(*p *< 0.05). PTA-S was not correlated with any bone rotation measure. PTA-G was positively correlated with femoral rotation, tibial rotation, and femoral rotation relative to tibia; while PTA-M was negatively correlated with femoral rotation, tibial rotation, and femoral rotation relative to tibia. (Table 2)

**Table 2:** Correlation between patella tilt angles and its neighboring bone rotations

	Patellar alignment					
	PTA-G		PTA-S		PTA-M	
Bone rotation						
FR	.737**	.000	-.106	.490	-.588**	.000
TR	.735**	.000	-.058	.705	-.377*	.011
FRRT	.270	.073	-.102	.506	-.494**	.001
	Pcc	*p *value	Pcc	*p *value	Pcc	*p *value

Pcc: Pearson correlation coefficient; Femoral rotation; TR: Tibial rotation; FRRT: Femoral rotation relative to tibia; PTA-G: Patella tilt angle of Grelsamer; PTA-S: Patella tilt angle of Sasaki; PTA-M: modified patella tilt angle of Fukerson.

Through regression analysis, curve estimation has demonstrated that femoral rotation, tibial rotation, and femoral rotation relative to tibia, as independent variables, served as significantly explanatory predictors in estimating the measures of PTA-G and PTA-M, as dependent variables. (Table 3 and Figures 2, 3, 4). The measure of PTA-G was more strongly predicted by femoral and tibial rotation, both exerted an R square of .54 (*p *< .01), in comparison to PTA-M, to which femoral rotation and tibial rotation exerted an R square of .35 and .14 respectively (*p *< .01 and .05). And as an independent variable, femoral rotation relative to tibia only showed a significant predictability in predicting PTA-M, with an R square of .24. (*p *< .01) In sharp contrast to PTA-G and PTA-M, PTA-S was rather inert to femoral rotation and tibial rotation with an R square of .01 or less. PTA-S has definitely isolated itself from the confounding effect of femoral and tibial rotation.

**Table 3:** Statistic values of regression analysis by curve estimation of patella tilting via its neighboring bone rotation

		R square	Beta	T	Sig T
Independent variable	Dependent variable				
Femoral rotation	PTA-G	.54	.74	7.16	.0000
	PTA-S	.01	-.11	-.70	.4899
	PTA-M	.35	-.59	-4.77	.0000
Tibial rotation	PTA-G	.54	.74	7.11	.0000
	PTA-S	.00	-.06	-.38	.7052
	PTA-M	.14	-.38	-2.67	.0106
Femoral rotation relative to tibia	PTA-G	.073	.270	1.84	.0731
	PTA-S	.01	-.10	-.67	.5062
	PTA-M	.24	-.49	-3.73	.0006

PTA-G: Patella tilt angle of Grelsamer; PTA-S: Patella tilt angle of Sasaki; PTA-M: modified patella tilt angle of Fukerson.

## Discussion

The current study has demonstrated various effects of regional bony alignment on the different measurements of the patellar tilt. The influence of femoral, tibial rotation, or femoral rotation relative to tibia on patella tilting varied with the difference in the way of measuring the patella tilt angle. PTA-G increased with increase in femoral, tibial rotation, or femoral rotation relative to tibia. PTA-S was stationary with any change in femoral, tibial rotation, or femoral rotation relative to tibia. While PTA-M decreased with increase in femoral, tibial rotation, or femoral rotation relative to tibia. As thus 2 of the 3 measurements of patellar tilting, PTA-G and PTA-M, failed to isolate patellar tilting from the confounding effect of its neighboring bone rotation (femoral, tibial rotation, or femoral rotation relative to tibia.) in people sustaining patellofemoral pain (PFPS). On the other side, among the 3 parameters in the current study, PTA-S has been demonstrated to be effective in isolating itself from the neighboring bone rotation in expressing the patellar alignment relative to the femur independent of its neighboring bone rotation. The clinical relevance of the current study is apparent. The clinical implications are two folds. One is PTA-S might be the parameter in favor to represent the rotational deviation of the patella or rotational alignment of the patella independent of regional bone rotation. The other implication is that the problem of patellar malalignment can not be treated, separately, independent of the related limb alignment. The significant confounding effect of femoral, tibial rotation, or femoral rotation relative to tibia on the patella tilting, as demonstrated by PTA-G and PTA-M, has warranted interventions at controlling the hip, pelvic motion and ankle motion when treating the patients with PFPS.

As a rotational malalignment of the patella, patellar tilting is subjected to the influence of the neighboring bone rotation other than the simple inter-relationship between the patella and its immediate neighborhood, the patellar sulcus. Abnormal motions of the tibia and femur are believed to have an effect on patellofemoral mechanics and therefore PFPS. [13] Femoral internal rotation has been reported to be the primary contributor to lateral patellar tilt. [13,14] Both tibial and femoral motions have significant effects on the biomechanics of the patellofemoral joint. With tibial rotation, the prmary effect on the patella is rotational. This pattern of motion occurs as a result of the patella being fixed to the tibia via the patellar tendon. With femoral rotation, the predominant forces acting on the patella are the bony geometry and the peripatellar soft tissue restraints.[19]

The limitations of the current study are two folds. One is the probable overestimation of the close association between patella tilting and its neighboring bone rotation (femoral, tibial rotation, or femoral rotation relative to tibia) by the measures, PTA-G as well as PTA-M. Seriously speaking, it's a matter of close association between measures rather than between limb mechanics and inherent patellofemoral mechanics. The other limitation is the failure in addressing the condition in weight-bearing. It has been suggested that the patellofemoral joint kinematics during non-weight-bearing could be characterized as the patella rotating on the femur, while the patellofemoral joint kinematics during weight-bearing could be characterized as the femur rotating underneath the patella. Femoral and patellar rotations concomitantly contribute to the patellofemoral joint kinematics. In regard to patellar tilt, in the non-weight-bearing condition, lateral patellar tilt appears to be the result of the patella rotating laterally on a relatively horizontal femur. In the weight-bearing condition, however, it is evident that the amount of lateral patellar tilt is due to femoral internal rotation, as the patella remains relatively horizontal. [14] The current study design was executed during non-weight-bearing condition. Even though the close association between femoral rotation and patella tilting has helped witness the effect of the altered lower extremity mechanics on patellofemoral mechanics and therefore PFPS [13], the current study still failed to simulate the ideal contingency of weight-bearing. Further works are demanded to clarify a lot to know.

## Conclusion

The current study has demonstrated the influence of bony malalignment on the patellar tilt. The effect of femoral, tibial rotation, or femoral rotation relative to tibia on the patella tilting varied with the difference in the way of measuring the patella tilt angle. PTA-G increased with increase in femoral, tibial rotation, or femoral rotation relative to tibia. PTA-S was stationary with increase in femoral, tibial rotation, or femoral rotation relative to tibia. While PTA-M decreased with increase in femoral, tibial rotation, or femoral rotation relative to tibia. Among the 3 parameters in the current study, PTA-S has been demonstrated to be effective in isolating itself from the neighboring bone rotation, in expressing the patellar alignment relative to the femur. In other words, either PTA-G or PTA-M is confounded by the neighboring bony mechanism. The clinical implications are two folds. One is PTA-S might be the parameter in favor to represent the rotational deviation of the patella or rotational alignment of the patella, independent of regional bone rotation. The other implication is that the problem of patellar malalignment can not be treated, separately, independent of the related limb alignment. The later clinical implication has to be verified by further works, with a comprehensive evaluation of the various treatments of patellar malalignment.

## Competing interests

The author(s) declare that they have no competing interests.

## Authors' contributions

YFL carried out the computed tomography studies, participated in the data processing and drafted the manuscript. MHJ carried out necessary correction in the writing. DHL participated in the design of the study and performed the statistical analysis. CKC conceived of the study, and participated in its design and coordination. All authors read and approved the final manuscript.
